# Do preventive child examinations in general practice reduce the risk of overweight and obesity?

**DOI:** 10.1093/eurpub/ckac129.050

**Published:** 2022-10-25

**Authors:** S Heuckendorff, C Eggertsen, JL Thomsen, K Fonager

**Affiliations:** 1 Department of Social Medicine, Aalborg University Hospital, Aalborg, Denmark; 2 Department of Pediatrics, Aalborg University Hospital, Aalborg, Denmark; 3 Center for General Medicine, Aalborg University, Aalborg, Denmark; 4 Department of Clinical Medicine, Aalborg University, Aalborg, Denmark

## Abstract

**Background:**

The prevalence of children with overweight and obesity is increasing. General practitioners in Denmark follow children throughout early childhood via the preventive child health examinations. These examinations are offered to all children from birth to the age of five. Thus, the general practitioners have a unique opportunity for early tracing and identification of overweight and obesity, but the impact of the examinations are not examined. Therefore, the aim of this study was to examine the association between attending preventive child health examinations and the risk of overweight and obesity at the age of six both for the total pediatric population and within groups of vulnerable children such as children of parents with low educational level or low household income.

**Methods:**

A population-based birth cohort study was conducted including all Danish children born from 2000-2012 using the Danish nationwide registers. Data included information on child participation in preventive health examinations at general practice, height and weight at the age of six, and parental information on socioeconomic factors.

**Results:**

The analyses included 801,444 children. Attending preventive child health examinations were not associated with a lower risk of overweight at the age of six. A lower risk of obesity was seen in children attending the examinations, both in the general population (RR 0.71, 95% CI 0.66-0.76) and within vulnerable groups (low level of maternal education: RR 0.80, 95% CI 0.72-0.89), low household income (RR 0.79, 95% CI 0.72-0.87). The risk of obesity was greater in the vulnerable groups than in the not-vulnerable groups.

**Conclusions:**

Attending preventive child health examinations were associated with a lower risk of obesity at the age of six, but not overweight. This was seen for both the general pediatric population and within vulnerable groups. The lowest risk of obesity was seen in the not-vulnerable groups.

**Key messages:**

• The results indicated that attending preventive child health examinations in general practice reduced the risk of obesity at the age of six, but not the risk of overweight.

• The lowest risk of obesity was seen in the not-vulnerable groups attending the preventive child health examinations in general practice.

